# Short-term *in situ* shading effectively mitigates linear progression of coral-killing sponge *Terpios hoshinota*

**DOI:** 10.1371/journal.pone.0182365

**Published:** 2017-08-07

**Authors:** Thangadurai Thinesh, Ramu Meenatchi, Ramasamy Pasiyappazham, Polpass Arul Jose, Muthamizh Selvan, George Seghal Kiran, Joseph Selvin

**Affiliations:** 1 Department of Microbiology, School of Life Sciences, Pondicherry University, Puducherry, India; 2 School of Biotechnology, Madurai Kamaraj University, Madurai, India; 3 Department of Ecology and Environmental Science, School of Life Sciences, Pondicherry University, Puducherry, India; 4 Department of Food Science and Technology, School of Life Sciences, Pondicherry University, Puducherry, India; Academia Sinica, TAIWAN

## Abstract

The coral-killing sponge, *Terpios hoshinota* is a global invasive species that has conquered coral patches within a short span of time, which has led to a significant decline in living coral cover at various geographical locations. In this study, we surveyed the linear progression and impact of the *Terpios* invasion on live coral patches along Palk Bay, Indian Ocean, from August 2013 to August 2015. The field inventory revealed an extensive fatality rate of 76% as a result of *Terpios* outbreak. Experimental findings showed that symbiotic cyanobacteria act as a nutritional factory for the aggressive growth of *Terpios*. Shading hypothetically impairs the nutritional symbiont of the invasive species: the effect of sunlight on cyanobacterial biomass and its influence on *Terpios* progression over live coral patches was tested through *in situ* shading experiments. This study showed that artificial shading with cotton fabric could effectively mitigate sponge growth on live coral without affecting coral homeostasis.

## Introduction

The encrusting coral-killing sponge *Terpios hoshinota* outbreak causes significant declines in living coral cover in tropical coral reefs. After the first report on *Terpios* by Bryan in 1973 [1], the invasion has been theorized to be expanding its geographical range [2] and has caused mortality in many coral genera [3,4]. The geographical locations where such invasions have occurred include American Samoa, the Philippines, Japan [2], Taiwan [4,5], the Great Barrier Reef [6], Indonesia [7], and recently in Maldives [8], India [9] and Mauritius [10].

*Terpios hoshinota* is a very thin encrusting sponge (aggressive space competitor) actively overgrowing on stony corals [1, 11–12]. Once invasion begins, it expands its range because of i) its fast growing nature (two-fold higher growth rate than corals) on live coral, ii) its larvae-producing capacity (easily spread to nearby locations), and iii) its long-lasting nature in the reef environment (prevents coral larvae settlement) [1–2, 13]. The sponge has been reported to have a linear growth rate of 11.5–23 mm month^-1^ [14], which can impact coral reefs on the square kilometer scale [1]. Moreover, it seems to be progressively extending its global range, migrating westwards through the Indian Ocean [14,15]. Hence, acquiring the information about *Terpios* outbreak at unchecked geographical locations is crucial to understand its geographical expansion and its ecology to take up proper management decisions.*Terpios* is a photosynthetic sponge that requires sunlight-derived energy for survival, growth and reproduction [16]. Recent studies have revealed a higher abundance of photosynthetic symbionts in the mesohyl portion of the actively progressing *Terpios* sponge [17]. Hence, management action to halt the *Terpios* outbreak could be achieved through shedding of nutritional symbionts (cyanobacteria) that rely on sunlight for photosynthesis (food) [17]. We hypothesized that reduced sunlight irradiation could stop the progression of sponge solely dependent on cyanobacterial photosynthesis for its growth. In this study, an inventory of the *T*. *hoshinota* outbreak in Palk Bay, conducted along with *in situ* field experiments, aimed to halt *Terpios* progression without disturbing corals, using artificial shading to reduce sunlight irradiation over *Terpios* colonies.

## Materials and methods

### Study site

Palk Bay is situated in a shallow, flat basin with a maximum depth of 5 m and an average depth of 3 m between longitudes (situated between longitudes 79° 17' 40" E and 79° 8' E and at a latitude of 9° 17' N). Palk Bay comprises a narrow reef, running parallel to the shore located 250m to 500m away from the shore and is discontinuous. Reef diversity has been reported to be declining due to anthropogenic disturbances such as oil pollution, waste discharge, trap fishing, emerging coral diseases, and unknown factors because of its proximity to shore and unprotected status [18,19]. A survey carried out during 2007–2013 showed that coral cover particularly *Acropora* population was continuing to decline in the reef of Palk Bay [20]. Moreover, a more recent survey during 2014 stated a reduced coral cover in the study site near Mandapam north (9° 27' 70'' N, 79° 12' 52'' E) [21]. However, the reason behind the coral cover decline remains unknown, since the above-mentioned inventories were carried out at preliminary scale without linking continuous monitoring and biotic factors.

### Sample collection, morphological and molecular identification of *Terpios hoshinota*

Representative coral encrusting sponge samples were collected by scraping off the sponge tissue using sterile dissecting forceps and were snap frozen in liquid nitrogen for analysing the chlorophyll content and also for the identification of encrusting sponge. Morphological identification of *T*. *hoshinota* was performed by following the diagnostic characters described by Rutzler and Muzik [12]. Further, actively growing specimens of *T*. *hoshinota* (1 mm thick dark black in color) at the coral front were collected for extracting total genomic DNA using standard phenol-chloroform protocol with slight modification [22] (by incorporating a grinding step using liquid nitrogen to lyse the cells mechanically by repeated freeze-thaw cycles). Using the eukaryotic 18S rRNA gene primers, Euk-A (5′-AACCTGGTTGATCCTGCCAGT-3′) and Euk-B (5′-GATCCTTCTGCAGGTTCACCTAC-3′) with an expected amplicon size of 1780 bp, polymerase chain reaction (PCR) was performed using thermal cycler (Eppendorf).The reaction mixture (50 μl) comprised of the following: 1× Taq buffer, 2.5 mM MgCl_2_, 200 μM of each dNTPs, 50 ng of genomic DNA, 0.5 μM each of forward and reverse primers and 0.25 μl of Taq DNA polymerase (5U/μl). PCR programme was set with initial denaturation at 94°C for 2 min (one cycle), followed by 30 cycles of denaturation at 94°C for 1 min, primer annealing at 53°C for 45 sec and extension at 72°C for 1 min; final extension at 72°C for 5 min, and were stored at 4°C. The purified PCR product was sequenced by dideoxy Sanger’s method (Macrogen Inc. Korea). The sequence was compared with other sponge sequences by using NCBI megaBLAST algorithm (http://blast.ncbi.nlm.nih.gov/Blast.cgi) for their pair wise identities. Multiple alignments of these sequences were carried out by ClustalW 1.83 version of EBI (www.ebi.ac.uk/cgibin/ClustalW/) with 0.5 transition weight. Phylogenetic tree was constructed in MEGA 7.0 version (www.megasoftware.net) using Neighbor-Joining method with BLAST similarity search restricted to only *Terpios* taxa (taxid: 283537) for better understanding in the phylogeny.

### *In situ* inventory of invasion and ecological succession of *Terpios* and extinction of coral patches

In this study, a survey was conducted from August 2013 to August 2015 every four months to assess the outbreak of *T*. *hoshinota* in Palk Bay. Twenty 20 × 4 m line intercept transects were placed end-to-end, parallel to the reef crest, with a gap of at least 20 m between transects at the centre of the outbreak of the sponge at Palk Bay. Permanent buoys with permanent markers were placed at both ends of each transect to replicate the same transect at every four month interval. Total healthy (live portion of corals), and sponge-covered corals were measured during every field observation to calculate its abundance using the line intercept method [23]. The colonies were recorded as an autonomous mass of skeleton with the size of live tissue being above 20 cm in diameter. Linear regression analysis was performed to understand the relationship between live coral cover decline and *Terpios* abundance.

### *In situ* shading experiments

**Fig 1:**
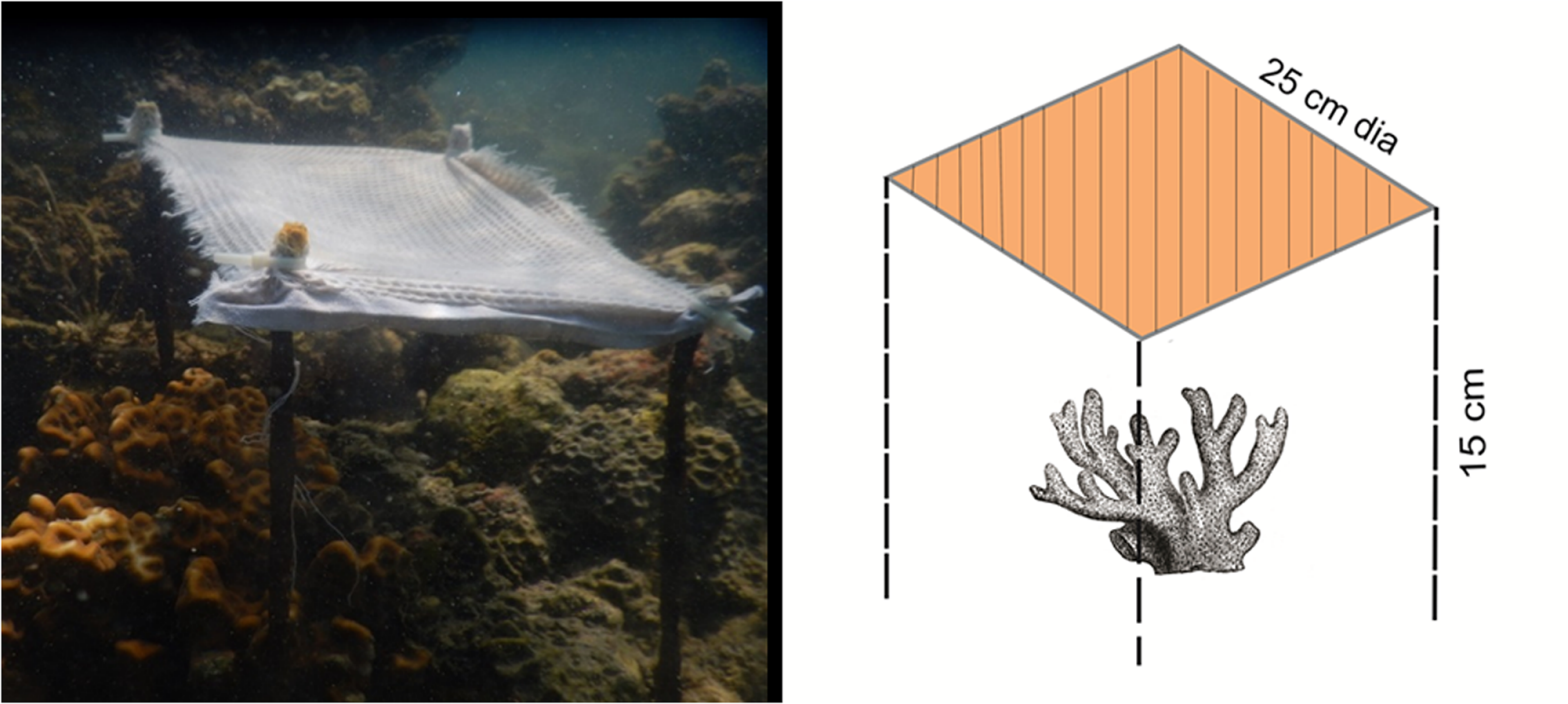
*In situ* experimental setup of temporary shading on coral colony, *Favites abdita* affected with *Terpios* sponge. Diagrammatic representation of the set up made is given on the right hand side.

In this study, the effects of sunlight on the progression of *Terpios* sponge over live coral colonies were recorded. The shading experiment was carried out in Palk Bay reef at 2 to 3 m depth (09° 20’ 052” N, 79° 17’ 468” E). *Favites abdita* colonies were selected for the experiment due to their suitability over *Acropora* sp. At the beginning of our experiment during May 2015, most of the *Acropora* colonies were nearly dead and consisted of uneven sample sizes. Further, the morphology of *F*. *abdita* facilitated quantitation of sponge progression, in comparison with the complexities associated with assessing the same in *Acropora* sp. (i.e.) *Acropora* is a branching coral species with uneven shape and morphology, whereas *F*. *abdita* is a massive coral species with comparatively smooth morphology and regular shape hence positively facilitates the tagging and monitoring process of *Terpios* progression. Ten colonies of *Terpios*-affected *F*. *abdita* from the same environment were tagged and randomly assigned to two treatments include shaded and unshaded at the same depth. In May 2015, five coral colonies were shaded using cotton lined fine white fabric (25 cm diameter) supported by four iron rods (Fig 1). The shading material was optimized *in vitro* and *in situ* using various fabric materials made up of wool, silk and cotton. The light penetration rates of the materials were assessed in the preliminary experiments. Light penetration (i.e. photosynthetically active radiation, PAR) was measured using Quantum meter (Lux-o-meter) [24] *in situ*. *In vitro*, the thickness of fabric used in the study was measured using fabric thickness gauge, as its thickness plays a key role in incidence of light on corals (which may in turn impact the coral homeostasis). The cotton lined fine white fabric was selected based on (i) effective filter of sunlight radiation up to 30%, (ii) biodegradable nature [25], and (iii) least absorbance of heat energy. Cotton being an organic material [25], which would naturally degrade in the event that the shade cloth became detached from its experimental set up.

**Fig 2:**
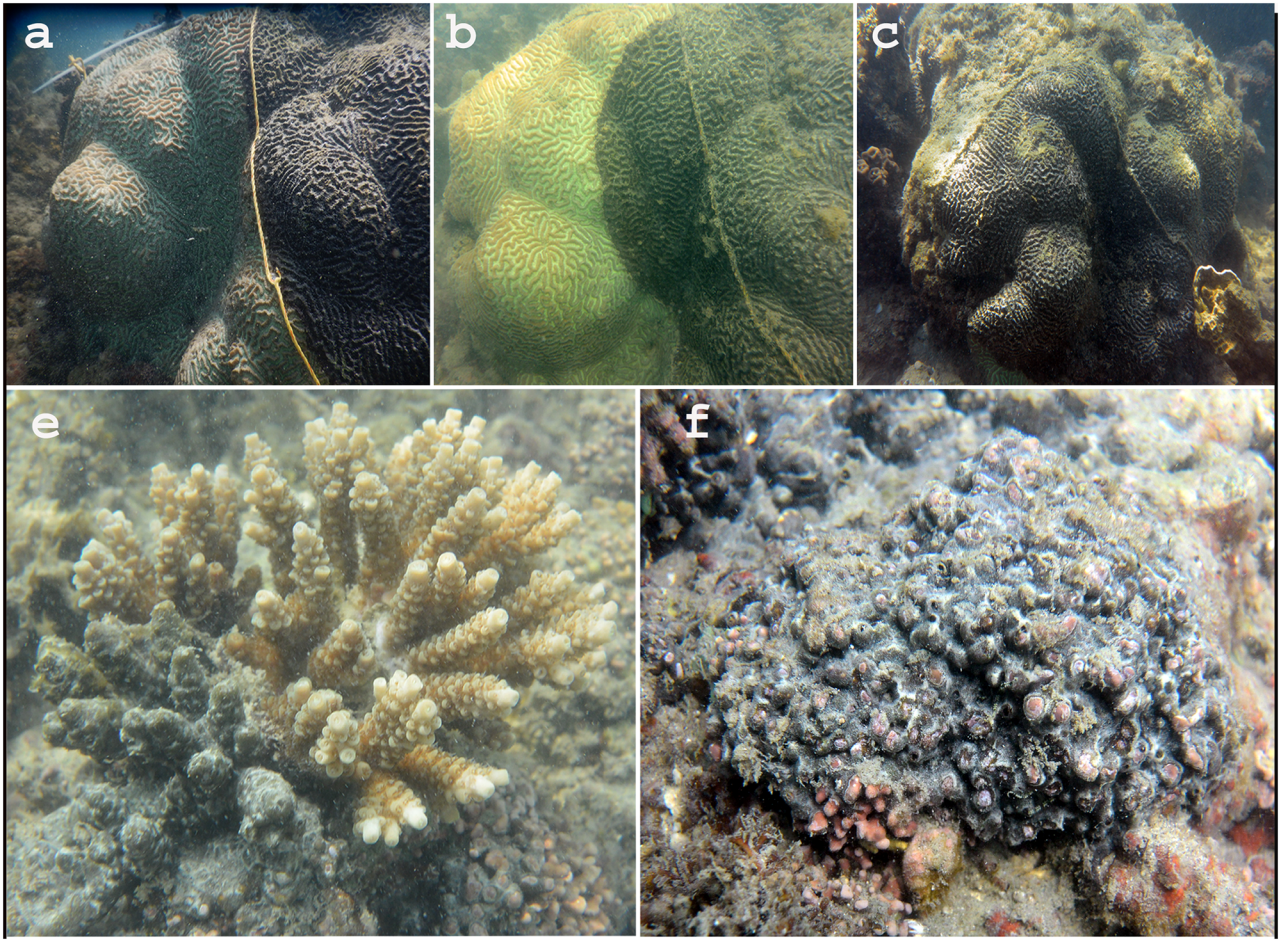
Linear progression of *Terpios* over live corals. Images *a*, *b* and *c* show *Terpios* progression over live coral *Platygyra* colony. Note the progression of *Terpios* over the tag. Images *d* and *e* show *Terpios* covered *Acropora* and coralline algae, respectively.

We used iron rods as a frame to set the fabric approximately 15 cm above the coral surface’s boundary layer to avoid stress through disruption of water flow. The idea was primarily aimed to allow partial sunlight needed for the coral to survive. Five coral colonies remained unshaded (i.e. control) to compare the effect of shading on *Terpios* linear progression. All the selected colonies were cable tied (tagged) at the active sponge tissue (1 mm thick, dark black in colour) appeared in the coral front (Fig 2) to measure the rate of sponge progression over coral colonies. Either side of the cable was fixed with the help of anchor placed at bottom of the sea carefully without disturbing the adjacent live corals. Active sponge tissue was closely observed and the distance from the tagged portion to the new sponge front was measured in both shaded and control coral colonies during every field observation. Based on preliminary experiments conducted for a period of 30 days, the observations were performed between 5^th^ and 10^th^ days of shading to note changes in *Terpios* growth. Following this, the distance from the tagged area to the new sponge front was measured every subsequent month for a period of six months using a flexible tape. Finally, after 12 months, ending in (i.e. May 2016), all experimental colonies were revisited to record the status of the coral colonies. Mann-Whitney U test was applied to determine the variation in progression rate between shaded and control coral colonies.

### Estimation of cyanobacterial symbiont abundance through chlorophyll *a* (Chl *a*) content in sponge

The Chl *a* concentration in the sponge tissue was reported to represent the corresponding cyanobacterial abundance in sponge biomass [16, 26]. During shading experiments, the cyanobacterial abundance in sponge biomass was determined by estimating the Chl *a* concentration using a spectrophotometric method adopted by Parsons et al. [27] with slight modifications. Briefly, the sponge sample (i.e. active front of sponge over corals) was collected from both shaded and control colonies and were weighed to determine the concentration (mg) of Chl *a* present in 1 g of sponge tissue. Weighed piece of lyophilized sponge was suspended in 8:2 acetone:water and kept overnight at 4°C. Each sample was centrifuged (2000 ×g, 15 min) to remove suspended solids, and the supernatant was transferred to a spectrophotometer cuvette for measuring the absorbance. Absorbance was measured at 662.6 and 645.6 nm (UV-Vis Spectrophotometer, Agilent). Chl *a* concentrations were estimated according to Parsons et al. [27] and were inferred as cyanobacterial abundance in sponge biomass. A Mann-Whitney U test was used to determine the variation in Chl *a* concentration between shaded and control coral colonies.

## Results

### Identification of coral encrusting sponge in Palk Bay

**Fig 3:**
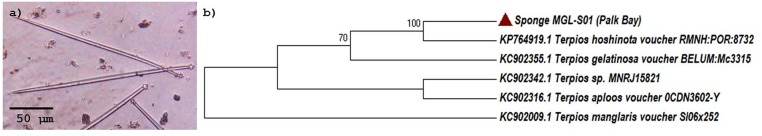
Identification of coral encrusting sponge collected from Palk Bay. a. Spicules of *T*. *hoshinota* showing lobed head of tylostyle spicules, b. Molecular phylogenetic analysis of *Terpios* sp. with related sponge species.

During *in situ* observations, it was found that the coral encrusting sponge progression appeared as a smothering black mat over coral colonies. Morphological identification of *Terpios* by microscopy showed the presence of tylostyle spicules. These tylostyles were long and slender pin-shaped, spicules with a typically developed head (tyle) consisting of four knobs with axes perpendicular to each other and to the shaft (Fig 3a). Phylogenetic analyses of 18S rRNA sequence (Fig 3b) showed that the coral encrusting sponge designated as MGL-S01 show 100% bootstrapping with *Terpios hoshinota*. Hence, the encrusting sponge from Palk Bay reef in India was named as *Terpios* sp. RM-2016, and the 18S rRNA sequence was deposited in NCBI database with an accession number KY171743.

The evolutionary history was inferred using the Neighbor-Joining method [1]. The bootstrap consensus tree inferred from 1000 replicates is taken to represent the evolutionary history of the taxa analysed. Branches corresponding to partitions reproduced in less than 50% bootstrap replicates are collapsed. The percentage of replicate trees in which the associated taxa clustered together in the bootstrap test (1000 replicates) are shown next to the branches. The evolutionary distances were computed using the Kimura 2-parameter method and are in the units of the number of base substitutions per site. The analysis involved 6 nucleotide sequences. All positions containing gaps and missing data were eliminated. There were a total of 611 positions in the final dataset. Evolutionary analyses were conducted in MEGA7.

### Impact of *T*. *hoshinota* on live coral cover

**Fig 4:**
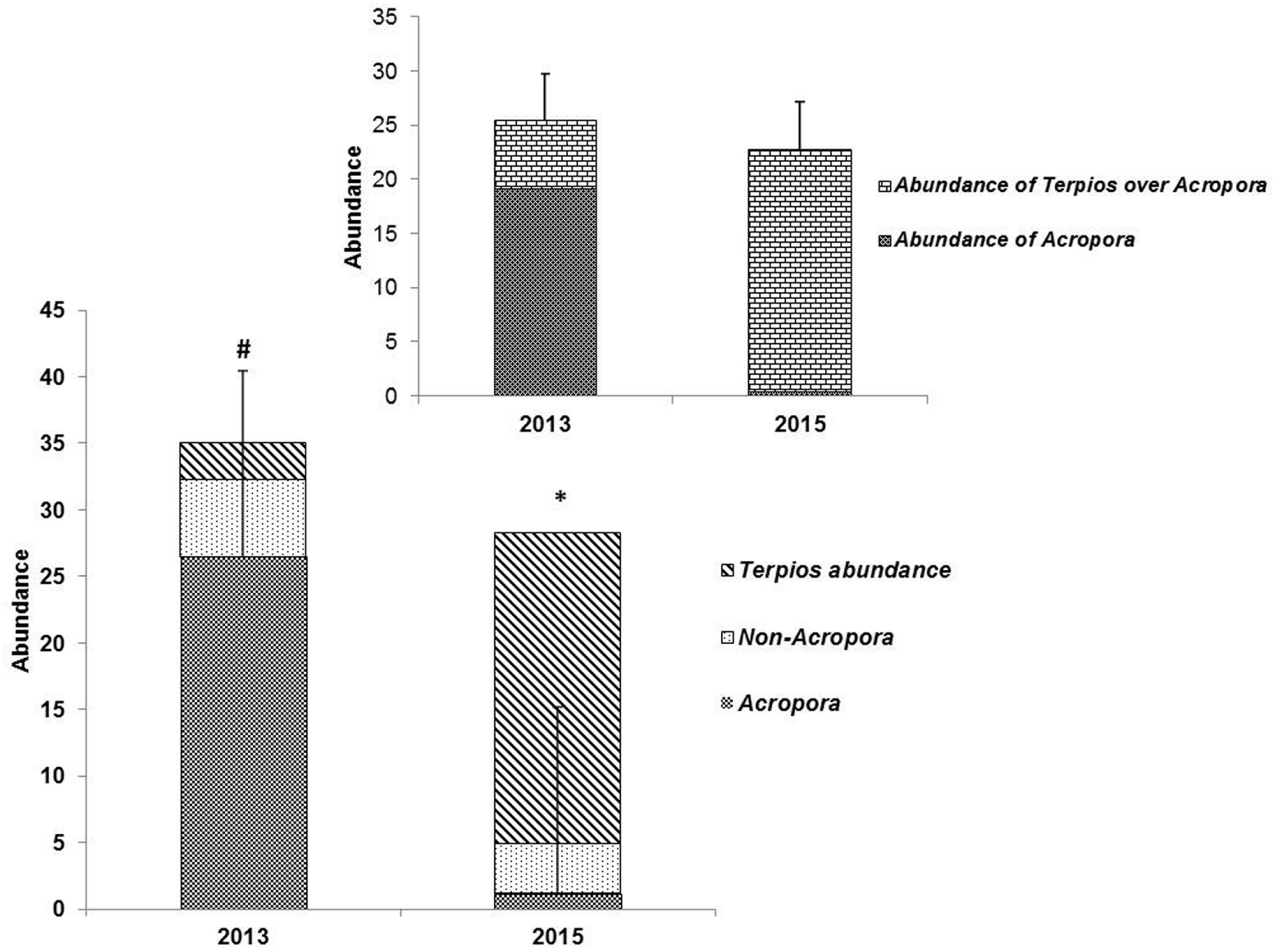
Average coverage of *Terpios* on coral colonies such as *Acropora*, Non-*Acropora* and *Terpios hoshinota* at the reef of Palk Bay during study period from August 2013 to August 2015. The * represents increase in abundance of *Terpios* within 2 years, and # represents the abundance of *Acropora* over the study site. Inset figure represents the *Terpios* overgrowth exclusively on *Acropora* colonies.

**Fig 5:**
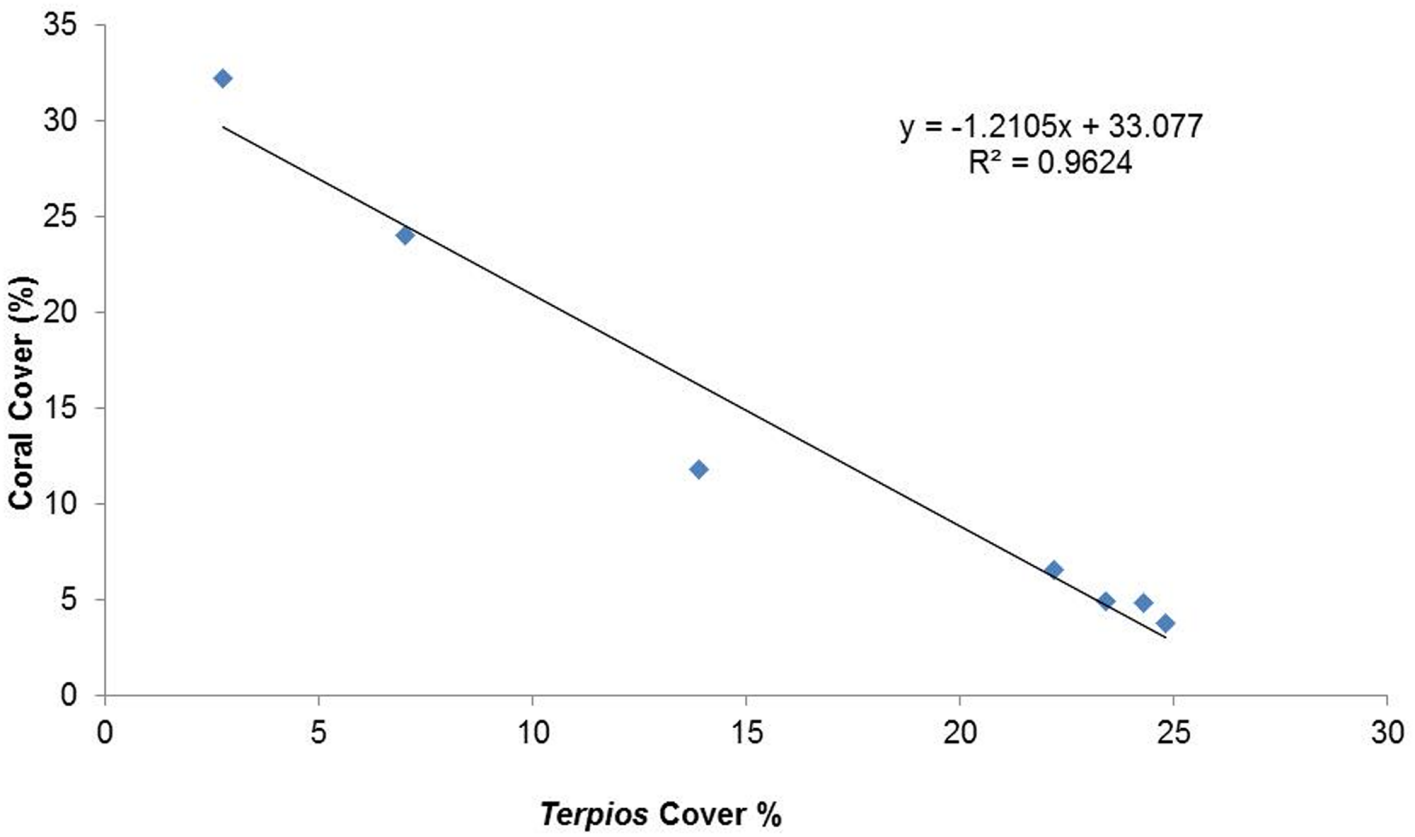
Linear regression between coral cover decline and *Terpios* abundance during the study period.

The field inventory data revealed that the invasive sponge *Terpios* caused extensive mortality in coral colonies in the study site, Palk Bay. At the beginning of our survey, mean live hard coral cover was 32.2 ± 5.5% in the total area surveyed along the transects, and was classified into two categories namely *Acropora* (26.4 ± 6.05%) and non-*Acropora* such as *Favites*, *Porites*, *Symphyllia*, *Favia*, *Goniastrea*, and *Platygyra* (5.8 ± 1.4%). Other encountered benthic categories were macroalgae and turf algae (25.2 ± 6.8%), hard substrates (21.2 ± 6.2%), sand (18.2 ± 6.2%), and seagrass and other sponges (3.1 ± 0.8%). Invariably, all coral patches were found to be covered with invasive sponge (Fig 2). Within two years of the survey period (August 2013 to August 2015), mean live coral cover was found to decline from 32.2 ± 5.5% to 3.41 ± 0.6%. The *Acropora* population was clustered together in the surveyed transect, while non *Acropora* was not found to be clustered in the study site. *Acropora* coral cover declined from 26.4 ± 6.05% to 0.42 ± 0.3%, while non-*Acropora* coral cover declined from 5.8 ± 1.4% to 3.32 ± 0.6%. *Terpios* abundance increased from 2.7% to 24.8% during the survey period from August 2013 to August 2015 (Fig 4). Linear regression analyses showed the relationship between coral cover decline and increased *Terpios* abundance (Fig 5). The overall survey data (7 surveys) depicting the coral cover and *Terpios* abundance is shown in supplementary S1 Fig.

### Optimization of shading material

Among the various fabric materials tested *in vitro* and *in situ*, cotton was selected based on effective filter of sunlight and least absorbent of heat energy as compared to wool and silk. The thickness of cotton fabric was 2 mm which was moderate as compared to wool and silk thickness were 5 mm and 0.2 mm, respectively. In the *in situ* experiments, the light transmittance through cotton was about 7956 (±2540) lux whereas, silk had comparatively more transmittance of light (19847 (±2341) lux). Wool was a thick fabric showed very low transmittance of light, 1750 (±1975) lux). Hence, cotton was selected for the shading experiments.

### Morphological appearance and progression rate of *Terpios* between control and *in situ* shaded coral colonies

**Fig 6:**
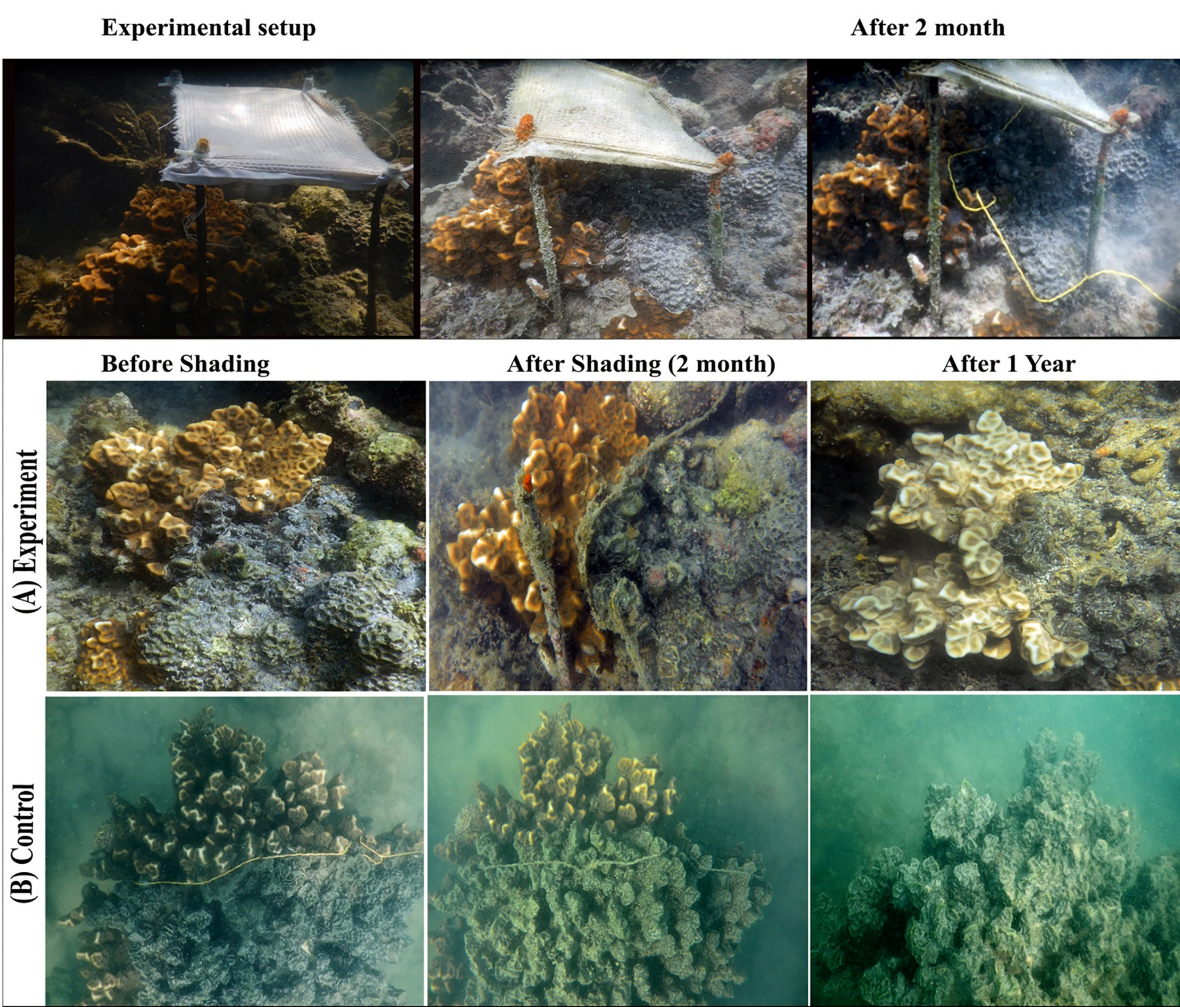
*In situ* shading experiment in Palk Bay. (A) Experimental colony after one year (*Terpios* progression was not observed). (B) Completely dead coral colony (fully invaded by *Terpios* within one year).

**Fig 7:**
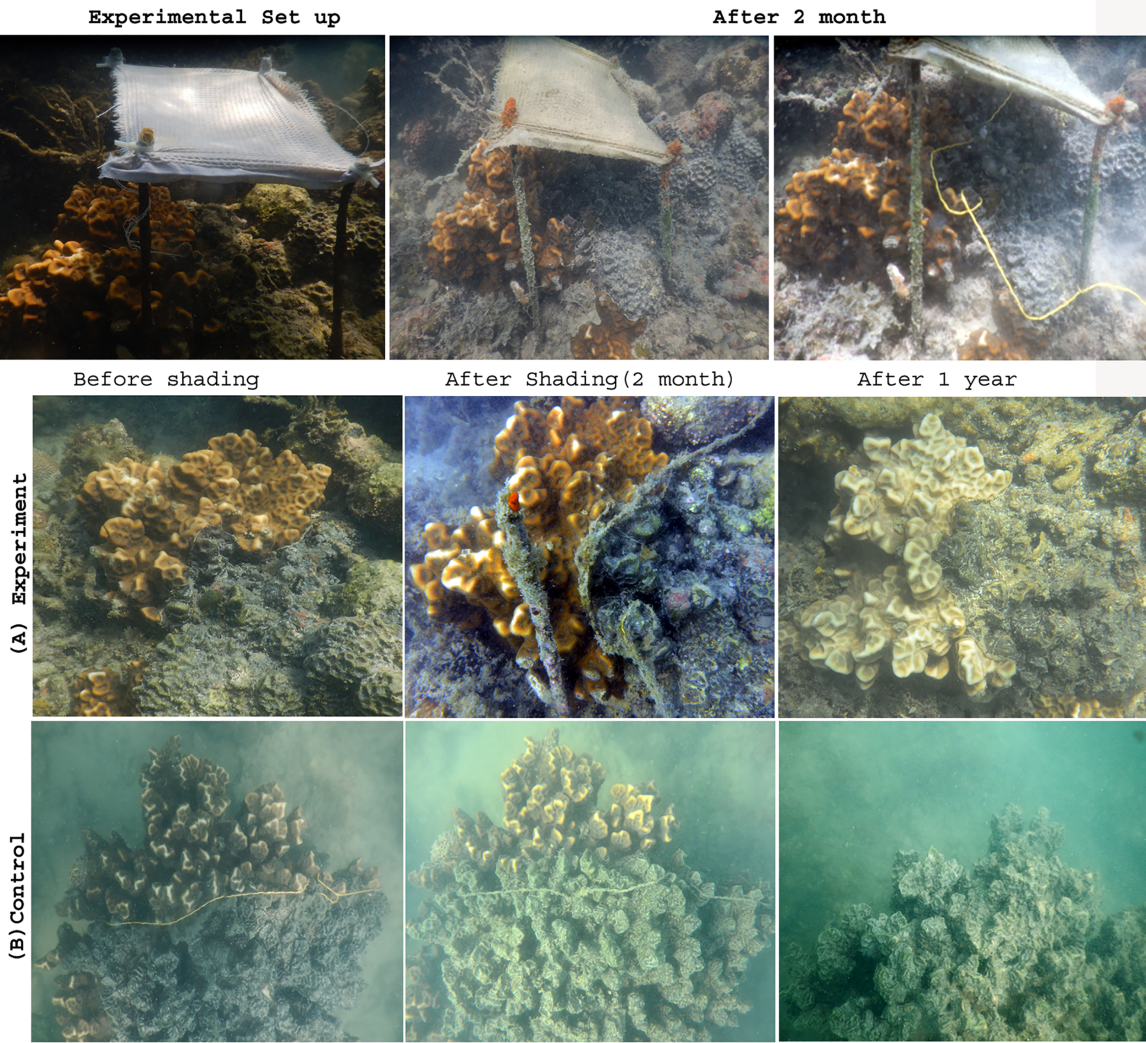
(a) Mean chlorophyll *a* content, (b) Progression rate difference between control and experimentally shaded coral colonies.

Field observations showed clear morphological differences between shaded and control *Terpios* sponges (Fig 6). In the control colonies, the observed *Terpios* smothering mat was brownish-black in color; the active band (immediately adjacent to corals) was 1 mm thick, and recently dead colonies were pale white in color. *In situ* shading showed a significant difference between experimental and control *Terpios*-covered coral colonies (Mann-Whitney U test, *p* < 0.05). Mean linear progression rate (19.4 ± 0.74 mm month^-1^) of *Terpios* on live coral colonies was observed in the control colonies (Fig 7b), whereas progression at a rate of 3.2 mm month^-1^ was noted in the shaded colonies. In shaded colonies, on 5^th^ day, no apparent change was observed while the disappearance of the 1 mm thick *Terpios* smothering mat was recorded on 10^th^ day of shading. Variation in sponge progression between shaded (3 mm) and control colonies (6 mm) was visible after 10^th^ day of shading. After a short-term shading exposure (10 days), the shading material was removed to observe whether *Terpios* regains active progression on coral colonies. Subsequent field observations were continued for six months colonies to check the status of experimental coral colonies. Finally, after 12 months, ending in May 2016, all experimental colonies had been revisited to record the status of the coral colonies.

Observations made after a month showed no signs of *Terpios* regeneration confirmed there was no active growth / progression of *Terpios* over the shaded colonies. Further, observations made over a period of six months and final observation after 12 months revealed the colonies that received short-term shading (10 days) were not having any signs of regeneration of *Terpios* on coral colonies. Regeneration at this point refers to active progression of sponge (i.e.) presence of thick black active sponge front that appeared during *Terpios* progression. During the post-shading observation period, we found no sign of *Terpios* regeneration and invasion over live coral colonies exposed for short-term shading. This observation showed the *Terpios* invasion was solely depend on cyanobacterial biomass accumulated in the black smothering mat. Hence, the short-term shading was effective to shed off the cyanobacterial symbionts irreversibly.

### Variation in cyanobacterial biomass

We found significant variation in the photosynthetic pigment, Chl *a* content between sponge samples collected from control and shaded colonies (Mann-Whitney U test, *p* < 0.05). On the 10^th^ day of the experiment, Chl *a* content in the control colonies was found to be 8.52 ± 0.153 mg g^-1^ as compared to the shaded colonies (3.34 ± 0.112 mg g^-1^, *p* < 0.05), thus indicating significant reduction as shown in Fig 7a. The Chl *a* content was also found to reduce continuously even after the post-shading experimental period (0.66 ± 0.122 mg g^-1^ on 30^th^ day post-shading).

## Discussion

### Case fatality rate

In recent decades, outbreak of coral killing sponge, *Terpios hoshinota* is frequently expanding its geographical range and is emerging as a threat to one of the most important coral reef ecosystems worldwide (30 to 80% mortality in coral reefs of various geographical locations) [1–10]. This is the first study to report evidence of rapid coral mortality in Palk Bay caused by *Terpios* invasion.

In our study, the fatality rate caused by the *T*. *hoshinota* invasion on live coral has increased from 2% to 24.8% within two years (i.e. 2013–15). This percentage of mortality was higher than the bleaching mortality observed in Palk Bay by other researchers [20,28]. Increase in coral mortality during *Terpios* outbreak can be attributed to the fact that sponges display more stable photochemical efficacy than corals under thermal stress [29–31]. For instance, Palk Bay, a reef ecosystem under unprotected status, is prone to both environmental (climate change-driven coral bleaching) [28,32] and anthropogenic (nutrient in flow, trap fishing) [18,19] stresses. The onset of coral bleaching was due to increased sea surface temperature which would eventually pose further detrimental effects on corals [33] through outbreak of invasive sponges.

In the South China Sea, rapid coral mortality has been reported to be over 75% due to *Terpios* invasion [3]. Liao et al. [4] reported 12% coral mortality caused by a *Terpios* invasion in Green Island reef within a year; and the reports from Guam and Japan [1–2] presents evidence of a spread of a *T*. *hoshinota* invasion across different geographical locations, including lagoons around small islands. The spread can extend from a few small patches (< 30 cm in diameter) to extensive, large patches (> 50 cm in diameter) covering most of the available hard substrate [1–2]. Moreover, Elliot et al. [14] found that *T*. *hoshinota* increased its abundance by 5% over one month by asexual reproduction. This high rate of spread of *Terpios* on branching corals has been observed in Indonesia [7] and Mauritius [10]. In South Eastern Japan reef, Reimer et al. [34] reported that *Terpios* were sporadic in nature that can cause sudden outbreaks and also disappear but cause irreversible damage to corals. In this study, we found persistent outbreak during our entire study period which caused irreversible damage to coral colonies. In Palk Bay, a higher fatality rate was found in *Acropora* than in the non-*Acropora* corals. Furthermore, *Terpios* may impact other organisms in the ecosystem. Although we did not quantify the impact of *Terpios* on coralline algae (CCA), an important substrate for coral recruitment [35] in the study site, CCA was most often found to be covered by *Terpios* as reported in other geographical locations [2].

### *Terpios* progression

In this study, observed sponge tissue at the coral front was a 1 mm thick dark brown band. Reports from Japan and Taiwan previously showed that the dark brown appearance could be attributed to the presence of cyanobacteria associated with sponge [2,12]. We found linear progression of *Terpios* growth on all control live coral colonies, with a mean progression rate of 19 ± 0.14 mm month^-1^ (Fig 6b). Progression rates in the present study were higher than the reports from Mauritius (11.5 mm month^-1^) and lower than those from Guam (23 mm month^-1^). This variation in progression rate between geographical locations might be due to environmental factors such as sunlight intensity. All the examined affected *Terpios* invaded coral colonies were completely covered by *Terpios*, resulting in coral mortality within a year. According to Yu et al. [36], aggressive sponge overgrowth arrests the photosynthetic function of coral without giving chances of polyp regeneration.

### Effect of *in situ* shading on cyanobacterial biomass and *Terpios* progression

*In situ* shading experiments showed that *Terpios* progression was associated with a dark brown banded mat, characterizing the cyanobacterial biomass. Short-term shading effectively reduced Chl *a* content in the shaded colonies, whereas Chl *a* content remain intact in control colonies. A significant effect of short-term shading was observed as the disappearance of 1 mm thick active brown banded sponge tissue. This finding evident that *Terpios* growth was solely dependent on association with cyanobacteria. Regeneration of *Terpios* was not observed after shading, suggesting that short-term shading effectively impaired the nutritional symbiont and irreversibly reduced cyanobacterial biomass. However, the reason why there was no recruitment of new cyanobacterial associates in the remaining live *Terpios* tissue after shading requires further research into the holobiont shift in *Terpios* during shading. In this study, shading material was optimized to provide effective shading to impair the progression of *Terpios* without affecting coral colonies. Wang et al. [17] reported that the maximum photosynthetic rate of *Terpios* reaches at an irradiance of >400 μmol photons m^-2^s^-1^, which was equal to ~22000 lux (1 μmol photons m^-2^s^-1^ = 54 lux). The cotton fabric used in this study for shading allows only about 8000 lux (7956 lux) which seems to be comparatively very low. This lowering light irradiance due to shading may have impaired the photosynthetic production of *Terpios*-associated cyanobacteria, which in turn may have stopped the *Terpios* overgrowth on corals. Cotton fabric short term shading used in our study pose no signs of bleaching or other anomalies on tagged coral colonies.

Despite shading not being possible at large geographical scales, the shading strategy was has been successfully adapted to rescue critically endangered species from an emerging bleaching threat [37]. Findings of this study showed that the artificial shading effectively mitigate sponge growth on live corals without affecting coral homeostasis. Based on the present findings, biodegradable non-polluting shading materials with biosensor systems can be developed as a method to protect coral reef ecosystems from invasive sponges.

## Supporting information

S1 Fig Relationship between coral cover decline and increased *Terpios* abundance during the overall survey period (for every 2 months interval) from August 2013 to August 2015. (TIF)
