# Correction: Short-term *in situ* shading effectively mitigates linear progression of coral-killing sponge *Terpios hoshinota*

**DOI:** 10.1371/journal.pone.0187004

**Published:** 2017-10-19

**Authors:** Thangadurai Thinesh, Ramu Meenatchi, Ramasamy Pasiyappazham, Polpass Arul Jose, Muthamizh Selvan, George Seghal Kiran, Joseph Selvin

Fig 7 is incorrect. Please see the correct Fig 7 here.

**Fig 7:**
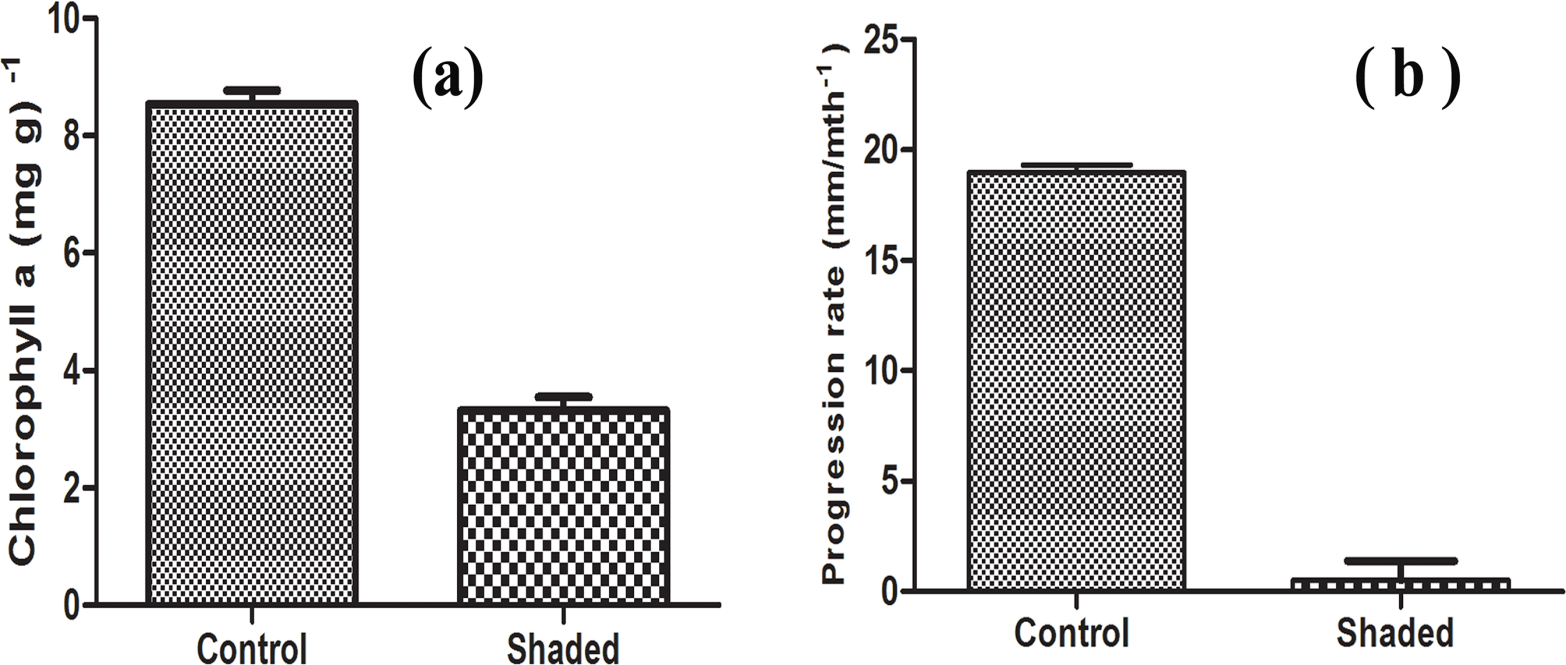
(a) Mean chlorophyll *a* content, (b) Progression rate difference between control and experimentally shaded coral colonies.
